# Pellagra From Tryptophan Depletion in Carcinoid Syndrome due to a Pulmonary Atypical Carcinoid Neuroendocrine Tumor

**DOI:** 10.1002/ccr3.71719

**Published:** 2026-03-25

**Authors:** Ahmar Iftikhar Talib, Cora Marks, Sheikh Saleem

**Affiliations:** ^1^ Department of Acute Medicine Arrowe Park Hospital Wirral UK

**Keywords:** lung neoplasms, malignant carcinoid syndrome, neuroendocrine tumors, nicotinamide, pellagra

## Abstract

Pellagra may complicate functioning pulmonary atypical carcinoid and present as a subtle acral dermatosis rather than the classical photo‐distributed rash. In neuroendocrine tumor patients with chronic diarrhea and new skin changes, clinicians should suspect niacin deficiency and consider empiric nicotinamide, even when niacin assays are unavailable.

## 1 Background

Neuroendocrine tumors (NETs) are rare neoplasms with variable clinical presentations. Carcinoid syndrome, characterized by diarrhea, flushing, and wheezing, results from excess serotonin secretion, most commonly from midgut NETs, although pulmonary cases are recognized. Excess serotonin synthesis from tryptophan can deplete precursors needed for niacin (vitamin B3) synthesis, potentially leading to pellagra, classically characterized by diarrhea, dermatitis, and dementia. Although now rarely seen in modern clinical practice, pellagra remains a potential complication of long‐standing carcinoid syndrome. Recognizing its often subtle cutaneous signs is key to early diagnosis and treatment.

## 2 Case History

Approximately 12 months before the dermatological presentation, a woman in her early 50s consulted her general practitioner with chronic non‐bloody diarrhea (3–6 episodes daily), episodic facial flushing, and abdominal discomfort. A fecal immunochemical test was positive, and she was urgently referred for colorectal assessment. Colonoscopy performed under the suspected cancer pathway revealed only benign polyps and hemorrhoids. Abdominal and pelvic ultrasonography revealed incidental small myometrial fibroids. Screening for infectious and coeliac disease was negative, and she was treated for presumed irritable bowel syndrome (IBS) with loperamide, mebeverine, and amitriptyline, with partial symptomatic relief. Over the following months, she experienced progressive weight loss of approximately 9 kg, fatigue, and back pain.

Several months before the dermatological presentation, she was referred to the acute oncology service with ongoing gastrointestinal symptoms. Cross‐sectional imaging of the thorax, abdomen and pelvis (CT TAP) revealed a 50‐mm left upper lobe lung mass with pulmonary artery invasion, effacement of the left upper lobe bronchus and lymphangitic spread, as well as bi‐lobar liver metastases and osseous lesions involving the right 2nd rib and T4 and T9 vertebral bodies.

Approximately a week later, the patient underwent endobronchial ultrasound‐guided aspiration (EBUS). Sampling of the mediastinal (4L) and hilar (11L) lymph nodes revealed atypical epithelial cells consistent with a neuroendocrine neoplasm, and subsequent immunohistochemistry revealed CD56 positivity and was negative for TTF‐1, SOX10, and p40. Twenty‐four‐hour urinary 5‐hydroxyindoleacetic acid (5‐HIAA) was markedly raised (644 μmol/24 h). An initial liver biopsy showed moderate hepatic steatosis without evidence of primary or metastatic malignancy. A subsequent CT‐guided lung biopsy confirmed atypical carcinoid tumor (synaptophysin and CD56 positive; Ki‐67 10%; mitotic figures present; no necrosis). Spirometry demonstrated preserved pulmonary function (FEV₁ and FVC both 102% predicted). The tumor was staged as T4N2M1c, and, in the absence of an alternative extrapulmonary primary, the lung lesion was considered the primary tumor.

Several weeks later, the patient was started on palliative lanreotide injections for carcinoid syndrome. Transthoracic echocardiography done a few weeks later showed preserved biventricular function, no regional wall motion abnormalities, and no evidence of carcinoid heart disease. DOTANOC PET‐CT and spinal MRI scans in the following weeks demonstrated widespread somatostatin receptor‐positive metastatic disease involving the lung, liver, and spine (at the T4, T8, T9, and probable L1 and L4 vertebral bodies). At clinic review around one month after starting lanreotide, she reported ongoing diarrhea, still up to 5 times daily. She was additionally taking pancreatic enzyme replacement (Creon).

Over a year after the onset of symptoms, and approximately 3 months after starting lanreotide, the patient presented to the acute medical unit with a five‐day history of a blanching, band‐like, erythematous rash over the dorsum of both feet. The rash was non‐pruritic, warm, and sharply demarcated, sparing the toes and plantar surfaces. There was no preceding trauma, insect bites, change in footwear, new medication, recent travel or significant sun exposure. She denied fever, malaise, arthralgia, joint stiffness, mucosal symptoms or acute neurological change. Flushing and diarrhea persisted but were unchanged from her baseline. She had trialed fexofenadine prior to presentation and had been compliant with the dietary plan advised by the dietitians.

**FIGURE 1:**
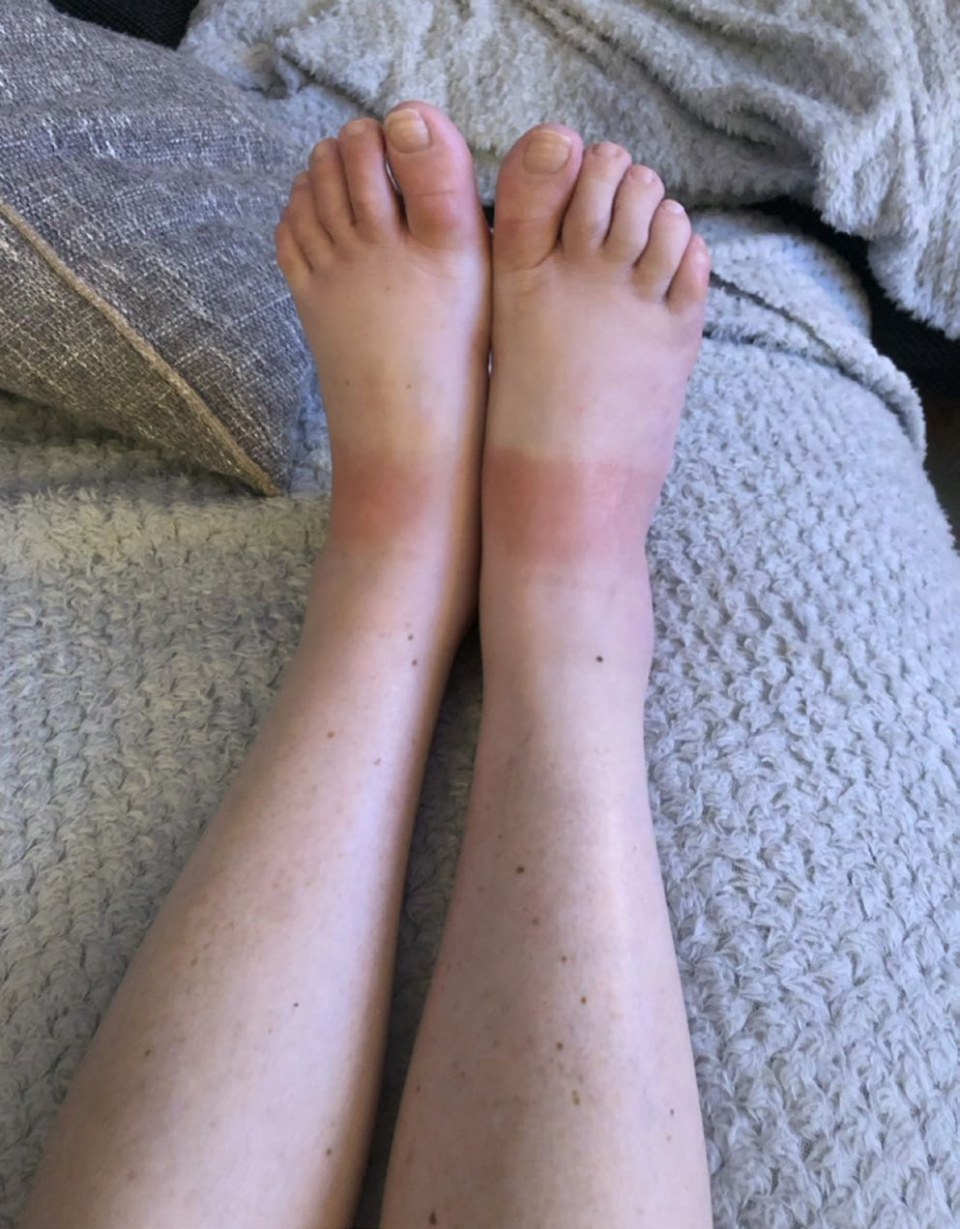
Bilateral sharply demarcated bands over the dorsal feet and ankles.

On examination, she remained systemically well. She was afebrile (37.0°C), with stable vital signs (blood pressure 100/61 mmHg, respiratory rate 18 breaths/min, heart rate 69 bpm, oxygen saturations of 98% on room air). Cardiovascular, respiratory, and abdominal examinations were unremarkable. Musculoskeletal examination revealed mild peri‐ankle swelling with full range of motion and no joint tenderness. Dermatological examination showed a sharply demarcated, blanching erythematous band across the dorsal aspects of both feet, with sparing of the toes and soles (Figure 1). The skin was warm but not tender, and there were no features suggestive of cellulitis or deep tissue infection.

Her past medical history included chronic lower back pain and depression. She was a lifelong nonsmoker, consumed approximately 10 units of alcohol per week, and worked as a teaching assistant. Her maternal aunt had been diagnosed with lung cancer at the age of 62.

## 3 Investigations, Differential Diagnosis and Treatment

Laboratory investigations at the time of the dermatological presentation are summarized in Table 1. Hemoglobin was 122 g/L (reference 115–165), white cell counts was 10 × 10^9^/L (4–11) and platelets 581 × 10^9^/L (150–400). C‐reactive protein was < 5 mg/L (< 5). Serum sodium was 134 mmol/L (135–145), potassium 4.1 mmol/L (3.5–5.3), urea 2.0 mmol/L (2.5–7.8) and creatinine 31 μmol/L (45–84). Liver biochemistry showed a cholestatic pattern with alkaline phosphatase 310 IU/L (35–104) and gamma‐glutamyl transferase 106 IU/L (12–43), with alanine aminotransferase 16 IU/L (10–35) and aspartate aminotransferase 26 IU/L (5–35) within normal limits. Adjusted calcium was 2.33 mmol/L (2.20–2.60). Vitamin B12 was elevated at 853 ng/L (191–663). Coagulation profile was normal. Recent twenty‐four‐hour urinary 5‐HIAA was markedly elevated at 644 μmol/24 h. Serum niacin was not measured, as this assay was not routinely available locally.

**TABLE 1:** Summary of laboratory investigations at the time of the dermatological presentation.

Test	Result	Reference range
Hemoglobin (Hb)	122 g/L	115–165
White cell count (WCC)	10 × 10^9^/L	4–11
Platelets	581 × 10^9^/L	150–400
CRP	< 5 mg/L	< 5
Sodium	134 mmol/L	135–145
Potassium	4.1 mmol/L	3.5–5.3
Urea	2.0 mmol/L	2.5–7.8
Creatinine	31 μmol/L	45–84
Alkaline phosphatase (ALP)	310 IU/L	35–104
Alanine transaminase (ALT)	16 IU/L	10–35
Aspartate aminotransferase (AST)	26 IU/L	5–35
Gamma‐glutamyl transferase (GGT)	106 IU/L	12–43
Adjusted calcium	2.33 mmol/L	2.2–2.6
Vitamin B12	853 ng/L	191–663
Coagulation profile	Normal	—
5‐HIAA (24 h urine)	644 μmol	< 47
Serum niacin	Not available	N/A

The rash's bilateral, sharply demarcated distribution, absence of systemic features, and normal inflammatory markers made cellulitis, erysipelas, and deep vein thrombosis unlikely. There was no history of new footwear, topical agents, or occupational exposures to suggest irritant or allergic contact dermatitis, and the clinical appearance was not suggestive of erythema multiforme, vasculitis, or other common erythematous dermatoses. In the context of serotonin‐secreting neuroendocrine tumor with chronic diarrhea, significant weight loss, low urea and creatinine consistent with reduced protein and muscle mass, and markedly elevated 5‐HIAA, nutritional dermatoses were strongly considered.

The band‐like, photo‐sparing erythema over acral extensor surfaces, together with her systemic and biochemical profile, was felt most consistent with pellagra secondary to tryptophan depletion from a serotonin‐producing atypical carcinoid tumor. A therapeutic trial of oral nicotinamide was therefore initiated at over‐the‐counter dosage after counseling regarding the potential adverse effects including flushing, headache, and angioedema. She was advised to continue regular dietetic follow‐up, with optimisation of protein and micronutrient intake.

## 4 Outcome and Follow‐Up

**FIGURE 2:**
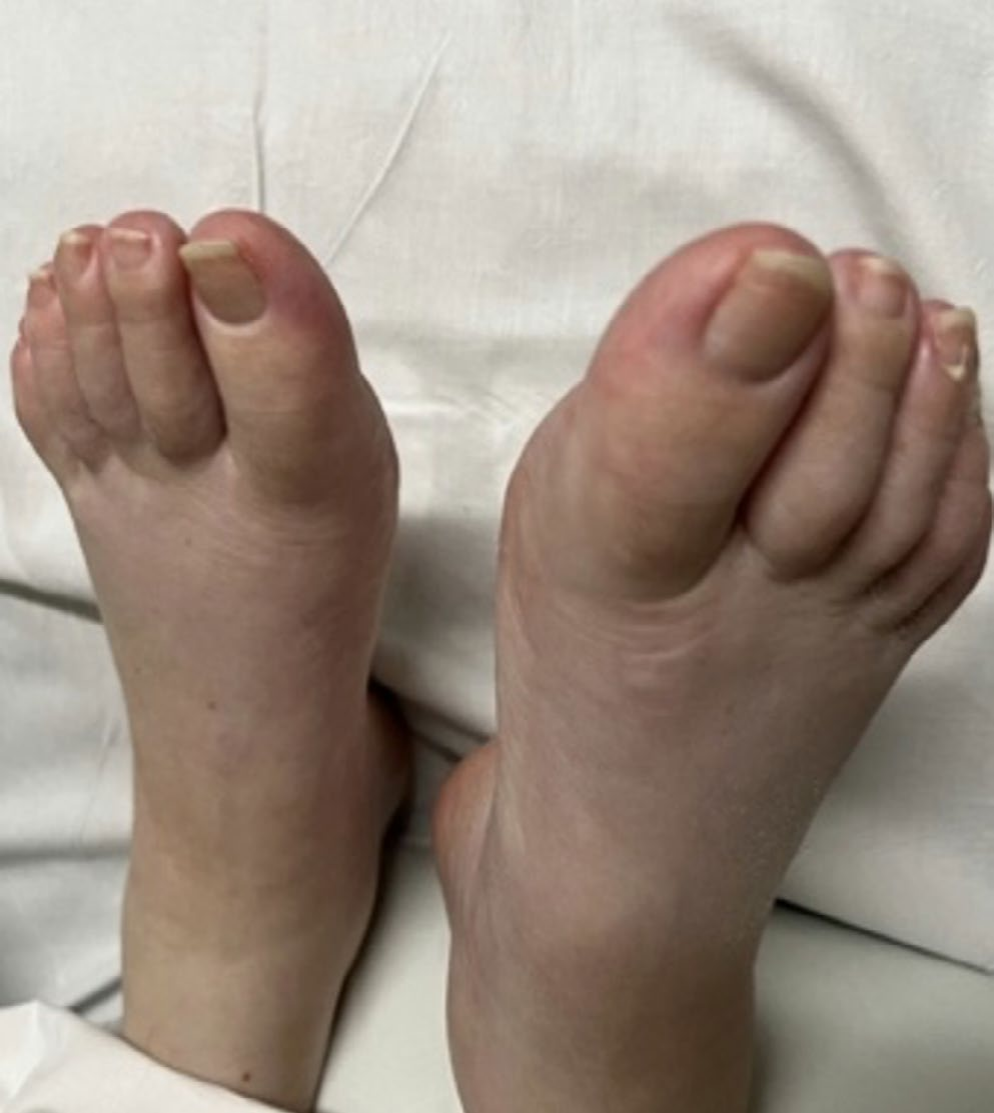
Resolution of the acral erythema following several weeks of nicotinamide supplementation.

Nicotinamide and multi‐vitamin supplementation were continued for several months. The erythematous eruption began to fade within weeks of starting therapy and resolved completely over the ensuing 2–3 months, with no recurrence reported (Figure 2). She experienced no adverse effects attributable to the nicotinamide. Despite resolution of the cutaneous manifestations, her metastatic neuroendocrine tumor continued to progress radiologically and clinically. She ultimately died from complications of advanced disease.

## 5 Discussion

Neuroendocrine tumors (NETs) are a heterogenous group of neoplasms arising from peptide‐ and amine‐secreting cells that possess dense‐core granules (DCGs) and the capacity to synthesize and release monoamines such as serotonin, bradykinin, calcitonin, and prostaglandins, as well as proteins such as chromogranin A [1]. These ultrastructural and functional features underpin their “neuroendocrine” designation, despite considerable variation in site of origin, presentation, grade, and clinical behavior [2].

Neuroendocrine cells are distributed widely throughout the body and were classically categorized by Williams and Sandler according to embryological origin into foregut (thymus, esophagus, lung, stomach, duodenum and pancreas), midgut (appendix, ileum, caecum and ascending colon) and hindgut (distal colon and rectum). The majority of NETs arise within the gastrointestinal tract (approximately 62%–67%), followed by the lung (22%–27%), which together account for most cases worldwide [3, 4, 5].

Although NETs remain relatively rare and hold orphan disease status in the US, approximately 0.5% of new malignancies are attributable to NETs, and 12%–22% are metastatic at presentation [5]. Females have been reported to be around 2.5 times more likely than males to develop a NET, and while most cases arise sporadically, associations with multiple endocrine neoplasia type 1 have been observed. Smoking and alcohol consumption have not been observed to increase the incidence of NETs overall, though this association varies by subtype [5, 6]. Gastrointestinal NETs have been observed more commonly in African American populations, whereas bronchial NETs appear to predominate in Caucasian cohorts [7, 8].

Pulmonary neuroendocrine neoplasms (NENs) account for approximately 20% of all primary lung cancers and comprise four main histological subtypes, in order of worsening prognosis: typical carcinoid, atypical carcinoid, large cell neuroendocrine carcinoma (LCNEC), and small cell lung carcinoma (SCLC) [9]. Well‐differentiated pulmonary NETs (typical and atypical carcinoids) represent only a small fraction of this group; typical carcinoids account for around 8% of pulmonary NENs and tend to occur in younger patients [10] whereas atypical carcinoids, such as in our patient, account for only about 1% and are more strongly associated with smoking and older male patients [11, 12, 13]. LCNEC and SCLC together make up the remaining ~90% of pulmonary NENs and are high‐grade neuroendocrine carcinomas.

A minority of pulmonary NENs are hormonally active “functioning” tumors. In these cases, ectopic hormone or peptide secretion can lead to paraneoplastic syndromes such as Cushing's syndrome or the syndrome of inappropriate antidiuretic hormone secretion (SIADH). When secretion is predominantly serotonergic, typically from well‐differentiated pulmonary NETs, patients may develop classical carcinoid syndrome with flushing and diarrhea, as seen in our patient [14, 15].

Carcinoid syndrome is most commonly described in metastatic midgut NETs. In this setting, serotonin and other vasoactive mediators are produced by enterochromaffin cells of the diffuse neuroendocrine system in the gut, where serotonin is synthesized from dietary tryptophan. Under normal circumstances, these mediators are carried via the portal circulation to the liver, where monoamine oxidases inactivate these monoamines. Carcinoid syndrome develops when these mediators reach the systemic circulation either by (a) bypassing hepatic metabolism via liver metastases, (b) being produced by extra‐abdominal tumors whose venous drainage is systemic rather than portal, or (c) being secreted in quantities sufficient to overwhelm hepatic clearance [16]. Serotonin synthesis depends on the essential amino acid tryptophan, which also serves as a precursor for nicotinamide adenine dinucleotide (NAD+), the active co‐enzyme form of niacin, via the kynurenine pathway. In functioning NETs with a sustained serotonin overproduction, up to 99% of dietary tryptophan (compared to the usual ~1%) can be catabolized into serotonin and thus diverting substrate away from nicotinamide synthesis. This can result in secondary niacin deficiency, classically characterized by the “four Ds” of pellagra—dermatitis, diarrhea, dementia, and in severe cases, death [17]. Primary causes such as poor dietary intake of niacin and tryptophan have become uncommon in developed countries following improvements in nutritional standards. Other secondary causes include chronic alcoholism, malabsorption syndromes, medication such as isoniazid and inherited disorders such as Hartnup disease [18].

Pellagra secondary to carcinoid syndrome is well recognized but appears to be rare, and most published reports involve midgut primaries. Castiello et al. [18] described six patients with pellagrous skin changes in the context of carcinoid syndrome, five of whom had gastrointestinal primaries and one hepatic metastasis from an unknown primary, with no pulmonary cases reported. Bell et al. performed a retrospective review over a two‐year period, identifying 25 patients with carcinoid syndrome; primary tumors were found in 19 patients, of which only three were pulmonary. All three patients with lung primaries experienced flushing and other cutaneous manifestations (xerosis, lichen planus, and folliculitis), but none developed pellagra [16]. Koch and Grayson [19] reported a 72‐year‐old woman who developed scleroderma secondary to a metastatic distal ileal NET, and Reichman and Sobel [20] described a premenopausal woman with intestinal carcinoid tumor and liver metastases who presented with vulvovaginal pellagra and low serum tryptophan, responding well to nicotinamide. Taken together, these reports indicate that both pellagra and other paraneoplastic dermatoses are predominantly described in association with non‐pulmonary primaries, emphasizing the rarity of pellagra‐like cutaneous disease arising from a pulmonary atypical carcinoid, as in our patient.

Our case adds to this literature by illustrating an unusual cutaneous phenotype of pellagra in the setting of a pulmonary atypical carcinoid. Unlike most reported cases which involve midgut or intestinal primaries with photo‐exposed and often generalized pellagrous eruptions, our patient had a well‐differentiated pulmonary NET and developed a sharply demarcated acral dermatitis confined to the dorsal feet, without classical facial or flexural involvement. In addition, she lacked overt neuropsychiatric features of pellagra, and the diagnosis rested on the combination of a functioning serotonin‐secreting tumor, chronic diarrhea and weight loss, low urea and creatinine suggesting poor protein stores, and a clear clinical response to nicotinamide.

The case also underlines the diagnostic challenges of recognizing pellagra in patients with advanced NETs. The rash was non‐specific in distribution, confined to the feet and easily misattributed to cellulitis, venous disease, or drug reactions. Diarrhea had been present for many months and could reasonably be explained by carcinoid syndrome, somatostatin analogue therapy, and exocrine pancreatic insufficiency, rather than as a new nutritional complication. Serum niacin levels were not available locally, reflecting common real‐world constraints. In this context, the decision to treat empirically with nicotinamide was based on a synthesis of clinical findings and pathophysiology rather than a single diagnostic test, and the complete resolution of the eruption with vitamin replacement retrospectively supported the working diagnosis.

This report has several strengths and limitations. Strengths include the detailed temporal reconstruction of symptom evolution, oncological work‐up and dermatological presentation, as well as photographic documentation and clear evidence of response to targeted therapy. We also link the clinical findings to established mechanisms of tryptophan diversion and niacin deficiency in serotonin secreting NETs. However, as a single case, our observations cannot be generalized to all patients with pulmonary NETs. We were unable to measure niacin or its metabolites, precluding biochemical confirmation of deficiency, and no skin biopsy was performed because it was not required to guide management, limiting histopathological corroboration. Finally, the multifactorial nature of diarrhea in this setting means we cannot precisely quantify the relative contributions of carcinoid syndrome, treatment‐related effects, and nutritional deficiency.

Nevertheless, this case emphasizes that pellagra should be considered in patients with functioning NETs who develop new dermatoses, particularly in the context of chronic diarrhea and weight loss, even when the primary tumor is pulmonary rather than midgut. In such patients, a low threshold for empiric nicotinamide supplementation is reasonable, given the low risk and potential to reverse cutaneous and systemic manifestations of niacin deficiency. Early recognition of this treatable nutritional complication may improve quality of life in individuals already burdened by advanced neuroendocrine malignancy. Biochemical studies in carcinoid syndrome have demonstrated clinically relevant niacin deficiency and symptomatic benefit from replacement, leading some authors to recommend empirical nicotinamide supplementation in at‐risk patients, especially in countries where dietary niacin fortification is limited [17].

## 6 Conclusion

In summary, this case illustrates a rare presentation of pellagra in a patient with functioning pulmonary atypical carcinoid, manifesting as a localized acral dermatosis rather than classical photo‐distributed disease. Clinicians should maintain a high index of suspicion for niacin deficiency in NET patients who present with chronic diarrhea and new skin changes and adopt a low threshold for empiric nicotinamide supplementation given its low risk and potential to significantly improve symptoms.

## Author Contributions

**Ahmar Iftikhar Talib:** writing – original draft, writing – review and editing. **Cora Marks:** writing – original draft, writing – review and editing. **Sheikh Saleem:** supervision, writing – review and editing.

## Funding

The authors have nothing to report.

## Consent

Informed, written consent was obtained from the patient's next of kin for publication of this report, and accompanying images.

## Conflicts of Interest

The authors declare no conflicts of interest.

## Data Availability

Data sharing not applicable to this article as no datasets were generated or analyzed during the current study.
